# Tris[4-(dimethyl­amino)­pyridinium] hexa­kis­(thio­cyanato-κ*N*)ferrate(III) monohydrate

**DOI:** 10.1107/S1600536812049574

**Published:** 2012-12-08

**Authors:** Susanne Wöhlert, Inke Jess, Christian Näther

**Affiliations:** a Institut für Anorganische Chemie, Christian-Albrechts-Universität Kiel, Max-Eyth-Strasse 2, 24118 Kiel, Germany

## Abstract

In the title compound, (C_7_H_11_N_2_)_3_[Fe(NCS)_6_]·H_2_O, the Fe^III^ cation is coordinated by six terminal *N*-bonded thio­cyanate anions into a discrete threefold negatively charged complex. Charge balance is achieved by three protonated 4-(dimethyl­amino)­pyridine cations. The asymmetric unit consists of one Fe^III^ cation, six thio­cyanate anions, three 4-(dimethyl­amino)­pyridinium cations and one water mol­ecule, all of them located in general positions.

## Related literature

For general background to our work on the synthesis and characterization of coordination compounds based on trans­ition metal thio­cyanates and neutral *N*-donor co-ligands such as pyridine, see: Boeckmann & Näther (2011 ▶, 2012 ▶).
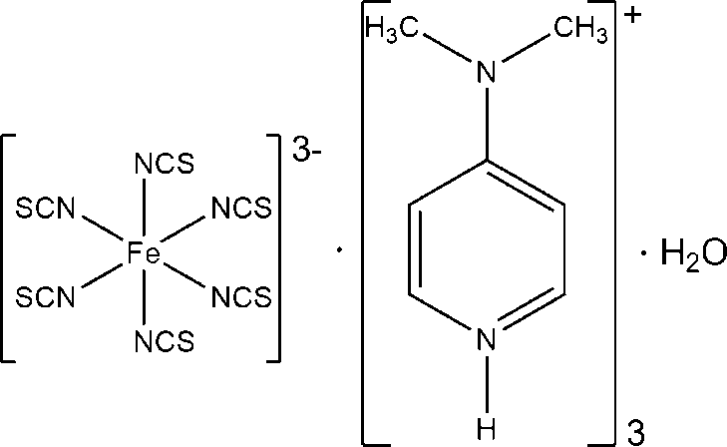


## Experimental

### 

#### Crystal data

(C_7_H_11_N_2_)_3_[Fe(NCS)_6_]·H_2_O
*M* *_r_* = 791.88
Triclinic, 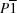

*a* = 11.5780 (7) Å
*b* = 11.7620 (7) Å
*c* = 16.5450 (11) Å
α = 81.260 (7)°
β = 71.550 (7)°
γ = 62.950 (6)°
*V* = 1903.4 (2) Å^3^
*Z* = 2
Mo *K*α radiation
μ = 0.77 mm^−1^
*T* = 180 K
0.13 × 0.08 × 0.06 mm

#### Data collection

Stoe IPDS-1 diffractometer
Absorption correction: numerical (*X-SHAPE* and *X-RED32*; Stoe & Cie, 2008 ▶) *T*_min_ = 0.808, *T*_max_ = 0.947
13617 measured reflections
7356 independent reflections
4936 reflections with *I* > 2σ(*I*)
*R*_int_ = 0.075

#### Refinement

*R*[*F* ^2^ > 2σ(*F* ^2^)] = 0.042
*wR*(*F* ^2^) = 0.104
*S* = 0.95
7356 reflections
431 parameters
H-atom parameters constrained
Δρ_max_ = 0.34 e Å^−3^
Δρ_min_ = −0.50 e Å^−3^

### 

Data collection: *X-AREA* (Stoe & Cie, 2008 ▶); cell refinement: *X-AREA*; data reduction: *X-AREA*; program(s) used to solve structure: *SHELXS97* (Sheldrick, 2008 ▶); program(s) used to refine structure: *SHELXL97* (Sheldrick, 2008 ▶); molecular graphics: *XP* in *SHELXTL* (Sheldrick, 2008 ▶) and *DIAMOND* (Brandenburg, 2012 ▶); software used to prepare material for publication: *XCIF* in *SHELXTL*.

## Supplementary Material

Crystal structure: contains datablock(s) I, global. DOI: 10.1107/S1600536812049574/hp2051sup1.cif

Structure factors: contains datablock(s) I. DOI: 10.1107/S1600536812049574/hp2051Isup2.hkl

Additional supplementary materials:  crystallographic information; 3D view; checkCIF report

## Figures and Tables

**Fig. 1.**
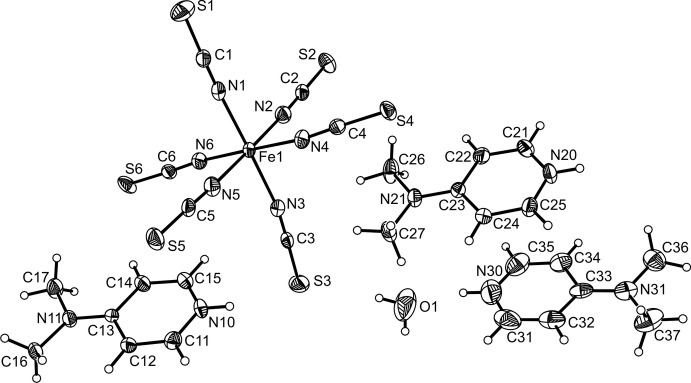
: Crystal structure of the title compound with labeling and displacement ellipsoids drawn at the 50% probability level.

**Fig. 2.**
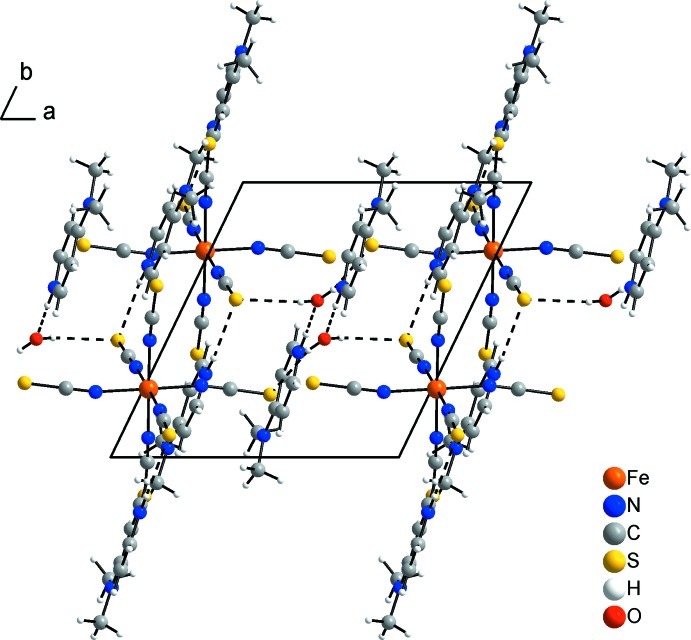
: Crystal structure of the title compound with view along the crystallographic c-axis. Intermolecular hydrogen bonding is shown as dashed lines.

## supplementary crystallographic information

### Comment

Recently we have reported on the synthesis and characterization of coordination compounds based on transition metal thiocyanates and neutral N-donor co-ligands like e.g. pyridine (Boeckmann & Näther, 2011, 2012). To investigate the influence of the co-ligand we tried to prepare similar compounds with N,N'-dimethylaminopyridine. Therefore, we have reacted iron(II) chloride tetrahydrate with potassium thiocyanate and N,N'-dimethylaminopyridine in water, which lead to the formation of single crystals of the title compound by accident. To identify the product of this reaction a single crystal structure determination was performed.

The crystal structure of the title compound consists of discrete [Fe(NCS)_6_]^3-^ anions, three N,N'dimethylaminopyridinium cations as well as one water molecule (Fig. 1). In the discrete complexes the iron(III) cations are coordinated by six thiocyanato anions within slightly distorted octahedra with distances in the range of 2.052 (2) Å to 2.079 (2) Å and angles ranging from 87.92 (10) ° to 91.80 (10) ° and from 178.35 (10) ° to 179.11 (10) ° (Tab. 1). The building blocks are connected via intermolecular N—H···S, N—H···O and O—H···S hydrogen bonding, in which the protonated N atom is involved (Tab. 2). These blocks are elongated in the direction of the crystallographic *a*-axis (Fig. 2).

### Experimental

FeCl_2_^.^4H_2_O and N,N'-dimethylaminopyridine were obtained from Sigma Aldrich. KNCS are obtained from Alfa Aesar. 0.3 mmol (59.6 mg) FeCl_2_^.^4H_2_O, 0.6 mmol (58.3 mg) KNCS and 0.15 mmol (18.3 mg) dimethylaminopyridine were reacted with 1 mL H_2_O in a snap cap vial. After one week red colored block-shaped single crystals of the title compound were obtained.

### Refinement

All C-H and N-H H atoms were located in difference map but were positioned with idealized geometry (methyl H atoms allowed to rotate but not to tip) and were refined isotropic with *U*_iso_(H) = 1.2 *U*_eq_(C, N) (1.5 for the methyl H atoms) using a riding model with C_aromatic_ = 0.95 Å, C_methyl_H = 0.98 Å and N—H = 0.88 Å. The O-H H were located in difference map, their bond lengths were set to ideal values and finally they were refined isotropic with *U*_iso_(H) = 1.5 *U*_eq_(O) using a riding model with O-H = 0.84 Å.

### Crystal data

**Table:**

(C_7_H_11_N_2_)_3_[Fe(NCS)_6_]·H_2_O	*Z* = 2
*M**_r_* = 791.88	*F*(000) = 822
Triclinic, *P*1	*D*_x_ = 1.382 Mg m^−^^3^
Hall symbol: -P 1	Mo *K*α radiation, λ = 0.71073 Å
*a* = 11.5780 (7) Å	Cell parameters from 13617 reflections
*b* = 11.7620 (7) Å	θ = 2.4–26.0°
*c* = 16.5450 (11) Å	µ = 0.77 mm^−^^1^
α = 81.260 (7)°	*T* = 180 K
β = 71.550 (7)°	Block, red
γ = 62.950 (6)°	0.13 × 0.08 × 0.06 mm
*V* = 1903.4 (2) Å^3^	

### Data collection

**Table:**

Stoe IPDS-1 diffractometer	7356 independent reflections
Radiation source: fine-focus sealed tube	4936 reflections with *I* > 2σ(*I*)
Graphite monochromator	*R*_int_ = 0.075
phi scan	θ_max_ = 26.0°, θ_min_ = 2.4°
Absorption correction: numerical (*X-SHAPE* and *X-RED32*; Stoe & Cie, 2008)	*h* = −14→14
*T*_min_ = 0.808, *T*_max_ = 0.947	*k* = −14→14
13617 measured reflections	*l* = −20→20

### Refinement

**Table:**

Refinement on *F*^2^	Secondary atom site location: difference Fourier map
Least-squares matrix: full	Hydrogen site location: inferred from neighbouring sites
*R*[*F*^2^ > 2σ(*F*^2^)] = 0.042	H-atom parameters constrained
*wR*(*F*^2^) = 0.104	*w* = 1/[σ^2^(*F*_o_^2^) + (0.052*P*)^2^] where *P* = (*F*_o_^2^ + 2*F*_c_^2^)/3
*S* = 0.95	(Δ/σ)_max_ = 0.001
7356 reflections	Δρ_max_ = 0.34 e Å^−^^3^
431 parameters	Δρ_min_ = −0.50 e Å^−^^3^
0 restraints	Extinction correction: *SHELXL97* (Sheldrick, 2008), Fc^*^=kFc[1+0.001xFc^2^λ^3^/sin(2θ)]^-1/4^
Primary atom site location: structure-invariant direct methods	Extinction coefficient: 0.0045 (11)

### Special details

**Table:**

Geometry. All esds (except the esd in the dihedral angle between two l.s. planes) are estimated using the full covariance matrix. The cell esds are taken into account individually in the estimation of esds in distances, angles and torsion angles; correlations between esds in cell parameters are only used when they are defined by crystal symmetry. An approximate (isotropic) treatment of cell esds is used for estimating esds involving l.s. planes.
Refinement. Refinement of F^2^ against ALL reflections. The weighted R-factor wR and goodness of fit S are based on F^2^, conventional R-factors R are based on F, with F set to zero for negative F^2^. The threshold expression of F^2^ > 2sigma(F^2^) is used only for calculating R-factors(gt) etc. and is not relevant to the choice of reflections for refinement. R-factors based on F^2^ are statistically about twice as large as those based on F, and R- factors based on ALL data will be even larger.

### Fractional atomic coordinates and isotropic or equivalent isotropic displacement parameters (Å^2^)

**Table:**

	*x*	*y*	*z*	*U*_iso_*/*U*_eq_	
Fe1	0.98269 (4)	0.75134 (4)	0.75439 (2)	0.01991 (12)	
N1	1.1616 (3)	0.7621 (3)	0.68821 (16)	0.0302 (6)	
C1	1.2706 (3)	0.7490 (3)	0.65317 (18)	0.0254 (6)	
S1	1.42277 (9)	0.72966 (11)	0.60257 (6)	0.0479 (2)	
N2	0.9072 (2)	0.8295 (2)	0.65142 (16)	0.0281 (5)	
C2	0.8775 (3)	0.8660 (3)	0.58809 (18)	0.0226 (6)	
S2	0.83393 (8)	0.91910 (8)	0.50092 (5)	0.0360 (2)	
N3	0.8051 (3)	0.7391 (3)	0.82393 (16)	0.0291 (6)	
C3	0.6995 (3)	0.7518 (3)	0.86786 (17)	0.0250 (6)	
S3	0.55149 (8)	0.77201 (10)	0.93009 (5)	0.0430 (2)	
N4	1.0601 (2)	0.5739 (2)	0.70384 (16)	0.0276 (5)	
C4	1.0898 (3)	0.4916 (3)	0.65919 (17)	0.0223 (6)	
S4	1.12768 (8)	0.37586 (7)	0.59908 (5)	0.02920 (18)	
N5	1.0629 (2)	0.6720 (2)	0.85664 (16)	0.0280 (5)	
C5	1.1078 (3)	0.6329 (3)	0.91418 (18)	0.0243 (6)	
S5	1.17023 (8)	0.57453 (8)	0.99515 (5)	0.0352 (2)	
N6	0.9021 (3)	0.9292 (2)	0.80598 (15)	0.0279 (5)	
C6	0.8648 (3)	1.0174 (3)	0.84754 (17)	0.0233 (6)	
S6	0.81802 (9)	1.14057 (7)	0.90350 (5)	0.0349 (2)	
N10	0.8102 (3)	0.6969 (2)	1.06431 (16)	0.0300 (6)	
H10	0.8080	0.6393	1.0374	0.036*	
C11	0.8352 (3)	0.6680 (3)	1.14068 (19)	0.0287 (6)	
H11	0.8516	0.5851	1.1642	0.034*	
C12	0.8376 (3)	0.7546 (3)	1.18468 (17)	0.0232 (6)	
H12	0.8539	0.7326	1.2390	0.028*	
C13	0.8157 (3)	0.8783 (3)	1.14994 (16)	0.0198 (5)	
C14	0.7897 (3)	0.9043 (3)	1.06899 (18)	0.0289 (6)	
H14	0.7731	0.9860	1.0432	0.035*	
C15	0.7884 (3)	0.8131 (3)	1.02853 (19)	0.0310 (7)	
H15	0.7719	0.8313	0.9742	0.037*	
C16	0.8466 (3)	0.9423 (3)	1.27260 (18)	0.0295 (6)	
H16A	0.7616	0.9703	1.3185	0.044*	
H16B	0.8965	0.9894	1.2756	0.044*	
H16C	0.9012	0.8507	1.2789	0.044*	
C17	0.7972 (4)	1.0927 (3)	1.1516 (2)	0.0345 (7)	
H17A	0.8679	1.0823	1.0977	0.052*	
H17B	0.8018	1.1450	1.1902	0.052*	
H17C	0.7085	1.1347	1.1407	0.052*	
N11	0.8174 (2)	0.9670 (2)	1.19072 (15)	0.0252 (5)	
N20	0.8066 (3)	0.2006 (3)	0.55337 (16)	0.0324 (6)	
H20	0.8113	0.1424	0.5231	0.039*	
C21	0.7879 (3)	0.3170 (3)	0.51934 (19)	0.0337 (7)	
H21	0.7819	0.3344	0.4625	0.040*	
C22	0.7775 (3)	0.4096 (3)	0.56454 (19)	0.0290 (6)	
H22	0.7631	0.4915	0.5395	0.035*	
C23	0.7880 (3)	0.3847 (3)	0.64942 (17)	0.0221 (6)	
C24	0.8093 (3)	0.2608 (3)	0.68218 (18)	0.0247 (6)	
H24	0.8173	0.2392	0.7385	0.030*	
C25	0.8183 (3)	0.1721 (3)	0.6331 (2)	0.0299 (7)	
H25	0.8331	0.0889	0.6556	0.036*	
C26	0.7592 (4)	0.6008 (3)	0.6599 (2)	0.0384 (8)	
H26A	0.8331	0.5927	0.6081	0.058*	
H26B	0.7588	0.6519	0.7017	0.058*	
H26C	0.6729	0.6429	0.6458	0.058*	
C27	0.8000 (3)	0.4446 (3)	0.78040 (19)	0.0327 (7)	
H27A	0.7285	0.4235	0.8192	0.049*	
H27B	0.7979	0.5188	0.8020	0.049*	
H27C	0.8880	0.3716	0.7768	0.049*	
N21	0.7785 (2)	0.4741 (2)	0.69564 (15)	0.0253 (5)	
N30	0.4772 (3)	0.3824 (4)	0.7836 (2)	0.0598 (10)	
H30	0.4722	0.4477	0.8067	0.072*	
C31	0.5092 (4)	0.2694 (5)	0.8242 (3)	0.0573 (12)	
H31	0.5254	0.2614	0.8781	0.069*	
C32	0.5190 (3)	0.1663 (4)	0.7905 (2)	0.0459 (9)	
H32	0.5446	0.0866	0.8199	0.055*	
C33	0.4912 (3)	0.1771 (3)	0.7116 (2)	0.0328 (7)	
C34	0.4594 (3)	0.2978 (3)	0.6703 (2)	0.0399 (8)	
H34	0.4428	0.3095	0.6162	0.048*	
C35	0.4524 (4)	0.3966 (4)	0.7073 (3)	0.0530 (10)	
H35	0.4298	0.4773	0.6792	0.064*	
C36	0.4696 (4)	0.0904 (4)	0.5952 (2)	0.0456 (9)	
H36A	0.5426	0.1023	0.5505	0.068*	
H36B	0.3832	0.1638	0.5954	0.068*	
H36C	0.4665	0.0124	0.5842	0.068*	
C37	0.5321 (4)	−0.0459 (4)	0.7194 (3)	0.0544 (10)	
H37A	0.4772	−0.0370	0.7790	0.082*	
H37B	0.6279	−0.0837	0.7172	0.082*	
H37C	0.5163	−0.1014	0.6898	0.082*	
N31	0.4946 (3)	0.0793 (3)	0.67822 (18)	0.0371 (6)	
O1	0.4555 (3)	0.5701 (4)	0.8757 (3)	0.0986 (15)	
H1O1	0.4927	0.6104	0.8866	0.148*	
H2O1	0.4004	0.5649	0.9218	0.148*	

### Atomic displacement parameters (Å^2^)

**Table:**

	*U*^11^	*U*^22^	*U*^33^	*U*^12^	*U*^13^	*U*^23^
Fe1	0.0247 (2)	0.0191 (2)	0.0198 (2)	−0.01218 (16)	−0.00614 (15)	−0.00240 (14)
N1	0.0341 (14)	0.0365 (15)	0.0283 (13)	−0.0208 (12)	−0.0074 (11)	−0.0083 (11)
C1	0.0323 (16)	0.0266 (15)	0.0241 (14)	−0.0169 (12)	−0.0104 (12)	0.0002 (11)
S1	0.0299 (4)	0.0642 (6)	0.0518 (5)	−0.0269 (4)	−0.0040 (4)	−0.0013 (5)
N2	0.0338 (13)	0.0265 (13)	0.0273 (13)	−0.0148 (11)	−0.0110 (11)	0.0017 (10)
C2	0.0217 (13)	0.0208 (14)	0.0264 (15)	−0.0090 (11)	−0.0067 (11)	−0.0044 (11)
S2	0.0438 (4)	0.0392 (5)	0.0268 (4)	−0.0141 (4)	−0.0178 (3)	−0.0017 (3)
N3	0.0332 (13)	0.0349 (15)	0.0273 (13)	−0.0215 (11)	−0.0065 (11)	−0.0043 (11)
C3	0.0336 (16)	0.0305 (15)	0.0203 (13)	−0.0187 (13)	−0.0124 (12)	−0.0001 (11)
S3	0.0322 (4)	0.0695 (6)	0.0321 (4)	−0.0292 (4)	−0.0023 (3)	−0.0044 (4)
N4	0.0339 (13)	0.0223 (12)	0.0309 (13)	−0.0154 (11)	−0.0085 (10)	−0.0027 (11)
C4	0.0268 (13)	0.0198 (14)	0.0214 (13)	−0.0117 (11)	−0.0074 (11)	0.0029 (11)
S4	0.0450 (4)	0.0209 (4)	0.0222 (3)	−0.0143 (3)	−0.0089 (3)	−0.0030 (3)
N5	0.0342 (13)	0.0249 (13)	0.0293 (13)	−0.0135 (11)	−0.0136 (11)	0.0004 (10)
C5	0.0279 (14)	0.0224 (14)	0.0271 (15)	−0.0139 (12)	−0.0066 (12)	−0.0058 (12)
S5	0.0428 (4)	0.0408 (5)	0.0301 (4)	−0.0193 (4)	−0.0193 (3)	0.0007 (3)
N6	0.0359 (13)	0.0257 (13)	0.0258 (12)	−0.0173 (11)	−0.0066 (10)	−0.0022 (10)
C6	0.0322 (14)	0.0204 (14)	0.0190 (13)	−0.0138 (12)	−0.0069 (11)	0.0020 (11)
S6	0.0600 (5)	0.0223 (4)	0.0203 (4)	−0.0178 (4)	−0.0078 (3)	−0.0022 (3)
N10	0.0403 (14)	0.0309 (14)	0.0256 (13)	−0.0194 (11)	−0.0085 (10)	−0.0081 (11)
C11	0.0323 (15)	0.0252 (15)	0.0304 (15)	−0.0148 (12)	−0.0068 (12)	−0.0013 (12)
C12	0.0280 (14)	0.0235 (14)	0.0202 (13)	−0.0132 (11)	−0.0074 (11)	0.0019 (11)
C13	0.0214 (12)	0.0244 (14)	0.0160 (12)	−0.0131 (11)	−0.0026 (10)	−0.0015 (10)
C14	0.0411 (16)	0.0313 (16)	0.0210 (14)	−0.0206 (14)	−0.0116 (12)	0.0031 (12)
C15	0.0397 (16)	0.0410 (18)	0.0215 (14)	−0.0240 (14)	−0.0113 (12)	0.0018 (13)
C16	0.0463 (17)	0.0311 (16)	0.0231 (14)	−0.0233 (14)	−0.0173 (13)	0.0042 (12)
C17	0.0528 (19)	0.0267 (16)	0.0351 (17)	−0.0228 (15)	−0.0206 (15)	0.0050 (13)
N11	0.0372 (13)	0.0233 (12)	0.0227 (12)	−0.0169 (11)	−0.0136 (10)	0.0027 (10)
N20	0.0379 (14)	0.0362 (15)	0.0299 (13)	−0.0198 (12)	−0.0073 (11)	−0.0119 (11)
C21	0.0413 (17)	0.046 (2)	0.0214 (14)	−0.0245 (15)	−0.0095 (12)	−0.0028 (13)
C22	0.0389 (16)	0.0320 (16)	0.0243 (14)	−0.0217 (14)	−0.0130 (12)	0.0068 (12)
C23	0.0216 (13)	0.0240 (14)	0.0226 (13)	−0.0119 (11)	−0.0049 (10)	−0.0020 (11)
C24	0.0274 (14)	0.0264 (15)	0.0215 (13)	−0.0136 (12)	−0.0055 (11)	−0.0001 (11)
C25	0.0332 (15)	0.0250 (15)	0.0342 (16)	−0.0156 (13)	−0.0060 (12)	−0.0051 (13)
C26	0.056 (2)	0.0208 (15)	0.050 (2)	−0.0160 (15)	−0.0328 (17)	0.0054 (14)
C27	0.0446 (18)	0.0310 (16)	0.0286 (15)	−0.0173 (14)	−0.0153 (13)	−0.0037 (13)
N21	0.0325 (12)	0.0214 (12)	0.0271 (12)	−0.0126 (10)	−0.0135 (10)	−0.0003 (10)
N30	0.0410 (18)	0.078 (3)	0.065 (2)	−0.0339 (18)	0.0046 (16)	−0.029 (2)
C31	0.039 (2)	0.098 (4)	0.037 (2)	−0.035 (2)	−0.0031 (16)	−0.005 (2)
C32	0.0353 (17)	0.067 (3)	0.0351 (18)	−0.0254 (18)	−0.0102 (14)	0.0108 (17)
C33	0.0196 (13)	0.0413 (18)	0.0308 (16)	−0.0127 (13)	−0.0040 (11)	0.0096 (13)
C34	0.0363 (17)	0.0393 (19)	0.0416 (19)	−0.0166 (15)	−0.0113 (14)	0.0073 (15)
C35	0.0395 (19)	0.047 (2)	0.068 (3)	−0.0205 (18)	−0.0082 (18)	0.002 (2)
C36	0.0387 (18)	0.050 (2)	0.043 (2)	−0.0155 (17)	−0.0080 (15)	−0.0057 (17)
C37	0.043 (2)	0.037 (2)	0.069 (3)	−0.0123 (17)	−0.0146 (18)	0.0185 (19)
N31	0.0301 (13)	0.0305 (14)	0.0393 (15)	−0.0082 (11)	−0.0050 (11)	0.0044 (12)
O1	0.066 (2)	0.123 (3)	0.122 (3)	−0.065 (2)	0.020 (2)	−0.064 (3)

### Geometric parameters (Å, º)

**Table:**

Fe1—N4	2.052 (2)	N20—H20	0.8800
Fe1—N6	2.059 (2)	C21—C22	1.353 (4)
Fe1—N2	2.061 (3)	C21—H21	0.9500
Fe1—N1	2.065 (3)	C22—C23	1.422 (4)
Fe1—N3	2.072 (3)	C22—H22	0.9500
Fe1—N5	2.079 (2)	C23—N21	1.338 (4)
N1—C1	1.157 (4)	C23—C24	1.413 (4)
C1—S1	1.618 (3)	C24—C25	1.365 (4)
N2—C2	1.165 (4)	C24—H24	0.9500
C2—S2	1.620 (3)	C25—H25	0.9500
N3—C3	1.162 (4)	C26—N21	1.461 (4)
C3—S3	1.625 (3)	C26—H26A	0.9800
N4—C4	1.164 (4)	C26—H26B	0.9800
C4—S4	1.621 (3)	C26—H26C	0.9800
N5—C5	1.162 (4)	C27—N21	1.464 (4)
C5—S5	1.634 (3)	C27—H27A	0.9800
N6—C6	1.165 (4)	C27—H27B	0.9800
C6—S6	1.620 (3)	C27—H27C	0.9800
N10—C11	1.345 (4)	N30—C31	1.339 (6)
N10—C15	1.347 (4)	N30—C35	1.351 (6)
N10—H10	0.8800	N30—H30	0.8800
C11—C12	1.353 (4)	C31—C32	1.355 (7)
C11—H11	0.9500	C31—H31	0.9500
C12—C13	1.419 (4)	C32—C33	1.415 (5)
C12—H12	0.9500	C32—H32	0.9500
C13—N11	1.336 (4)	C33—N31	1.330 (5)
C13—C14	1.423 (4)	C33—C34	1.418 (5)
C14—C15	1.356 (4)	C34—C35	1.354 (6)
C14—H14	0.9500	C34—H34	0.9500
C15—H15	0.9500	C35—H35	0.9500
C16—N11	1.454 (4)	C36—N31	1.467 (5)
C16—H16A	0.9800	C36—H36A	0.9800
C16—H16B	0.9800	C36—H36B	0.9800
C16—H16C	0.9800	C36—H36C	0.9800
C17—N11	1.466 (4)	C37—N31	1.457 (5)
C17—H17A	0.9800	C37—H37A	0.9800
C17—H17B	0.9800	C37—H37B	0.9800
C17—H17C	0.9800	C37—H37C	0.9800
N20—C25	1.344 (4)	O1—H1O1	0.8400
N20—C21	1.346 (4)	O1—H2O1	0.8401
			
N4—Fe1—N6	179.11 (11)	N20—C21—H21	119.3
N4—Fe1—N2	88.68 (10)	C22—C21—H21	119.3
N6—Fe1—N2	91.24 (10)	C21—C22—C23	120.1 (3)
N4—Fe1—N1	89.09 (10)	C21—C22—H22	119.9
N6—Fe1—N1	91.80 (10)	C23—C22—H22	119.9
N2—Fe1—N1	90.15 (10)	N21—C23—C24	121.8 (3)
N4—Fe1—N3	91.19 (10)	N21—C23—C22	121.6 (3)
N6—Fe1—N3	87.92 (10)	C24—C23—C22	116.6 (3)
N2—Fe1—N3	91.48 (10)	C25—C24—C23	120.1 (3)
N1—Fe1—N3	178.35 (10)	C25—C24—H24	119.9
N4—Fe1—N5	91.08 (10)	C23—C24—H24	119.9
N6—Fe1—N5	89.02 (10)	N20—C25—C24	121.1 (3)
N2—Fe1—N5	178.77 (10)	N20—C25—H25	119.5
N1—Fe1—N5	88.64 (10)	C24—C25—H25	119.5
N3—Fe1—N5	89.73 (10)	N21—C26—H26A	109.5
C1—N1—Fe1	170.0 (3)	N21—C26—H26B	109.5
N1—C1—S1	178.9 (3)	H26A—C26—H26B	109.5
C2—N2—Fe1	173.1 (2)	N21—C26—H26C	109.5
N2—C2—S2	178.9 (3)	H26A—C26—H26C	109.5
C3—N3—Fe1	168.7 (2)	H26B—C26—H26C	109.5
N3—C3—S3	178.9 (3)	N21—C27—H27A	109.5
C4—N4—Fe1	162.8 (2)	N21—C27—H27B	109.5
N4—C4—S4	178.3 (3)	H27A—C27—H27B	109.5
C5—N5—Fe1	176.9 (2)	N21—C27—H27C	109.5
N5—C5—S5	178.4 (3)	H27A—C27—H27C	109.5
C6—N6—Fe1	167.6 (2)	H27B—C27—H27C	109.5
N6—C6—S6	177.9 (3)	C23—N21—C26	120.9 (2)
C11—N10—C15	120.8 (2)	C23—N21—C27	121.1 (2)
C11—N10—H10	119.6	C26—N21—C27	117.7 (2)
C15—N10—H10	119.6	C31—N30—C35	120.3 (4)
N10—C11—C12	121.4 (3)	C31—N30—H30	119.9
N10—C11—H11	119.3	C35—N30—H30	119.9
C12—C11—H11	119.3	N30—C31—C32	121.8 (4)
C11—C12—C13	120.2 (3)	N30—C31—H31	119.1
C11—C12—H12	119.9	C32—C31—H31	119.1
C13—C12—H12	119.9	C31—C32—C33	120.1 (4)
N11—C13—C12	122.5 (2)	C31—C32—H32	120.0
N11—C13—C14	121.2 (3)	C33—C32—H32	120.0
C12—C13—C14	116.3 (2)	N31—C33—C32	121.8 (3)
C15—C14—C13	120.4 (3)	N31—C33—C34	121.9 (3)
C15—C14—H14	119.8	C32—C33—C34	116.3 (3)
C13—C14—H14	119.8	C35—C34—C33	120.4 (4)
N10—C15—C14	120.9 (3)	C35—C34—H34	119.8
N10—C15—H15	119.5	C33—C34—H34	119.8
C14—C15—H15	119.5	N30—C35—C34	121.1 (4)
N11—C16—H16A	109.5	N30—C35—H35	119.4
N11—C16—H16B	109.5	C34—C35—H35	119.4
H16A—C16—H16B	109.5	N31—C36—H36A	109.5
N11—C16—H16C	109.5	N31—C36—H36B	109.5
H16A—C16—H16C	109.5	H36A—C36—H36B	109.5
H16B—C16—H16C	109.5	N31—C36—H36C	109.5
N11—C17—H17A	109.5	H36A—C36—H36C	109.5
N11—C17—H17B	109.5	H36B—C36—H36C	109.5
H17A—C17—H17B	109.5	N31—C37—H37A	109.5
N11—C17—H17C	109.5	N31—C37—H37B	109.5
H17A—C17—H17C	109.5	H37A—C37—H37B	109.5
H17B—C17—H17C	109.5	N31—C37—H37C	109.5
C13—N11—C16	122.4 (2)	H37A—C37—H37C	109.5
C13—N11—C17	120.6 (2)	H37B—C37—H37C	109.5
C16—N11—C17	116.9 (2)	C33—N31—C37	121.7 (3)
C25—N20—C21	120.7 (3)	C33—N31—C36	121.3 (3)
C25—N20—H20	119.6	C37—N31—C36	116.8 (3)
C21—N20—H20	119.6	H1O1—O1—H2O1	105.9
N20—C21—C22	121.3 (3)		
